# Expanding the Assessment of Research Activity

**DOI:** 10.1007/s00062-020-00948-5

**Published:** 2020-09-03

**Authors:** László Solymosi

**Affiliations:** Dept. of Neuroradiology, Universitätsklinikum Würzburg, Würzburg, Germany

Traditionally, in our September issue we report on the development of our journal, following the official announcement of the Impact Factor in June. The Impact Factor is no longer considered the only gauge of the impact of journals around the world, including Springer Nature journals such as *Clinical Neuroradiology*. This year Springer Nature signed the Declaration on Research Assessment (DORA).

In accordance with DORA, we will inform you both in this Editorial as well as continuously on the journal homepage about *Clinical Neuroradiology*’s journal metrics and development. On the journal homepage, the following measures are displayed: the 2‑year Impact Factor, 5‑year Impact Factor, article downloads and article handling times. On an individual article page, readers are informed about accesses and citations to the article as well as the altmetric score, a measure of an article’s online visibility and impact.

**Fig. 1:**
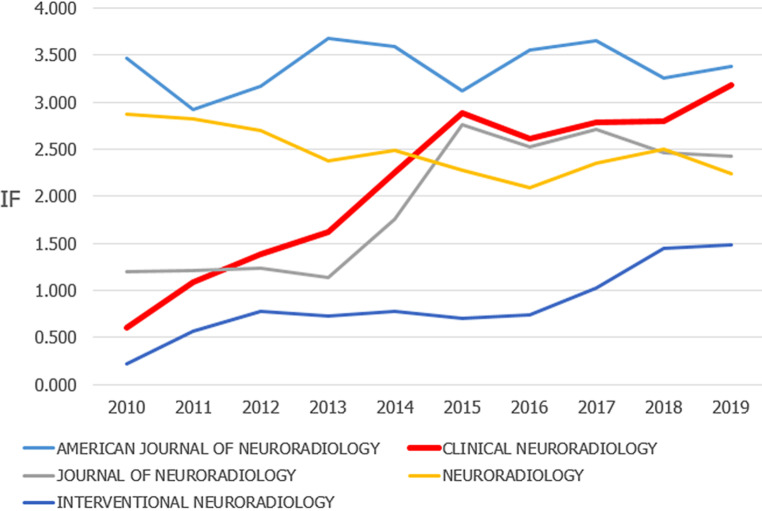
Impact Factor (IF) development of neuroradiological journals in the last 10 years

The development of *Clinical Neuroradiology* is very good: according to Clarivate, the 2019 Impact Factor has passed the 3.0 mark and is currently 3.183. This means that *Clinical Neuroradiology* maintains its second place among all neuroradiological journals and has even caught up to AJNR (Fig. 1).

In addition to its Impact Factor, *Clinical Neuroradiology* has also improved its rankings in the two subject categories in which it is indexed. In the category “Clinical Neurology”, the journal ranks 73rd of 204 journals, compared to 88th of 199 journals in the previous year. In the category “Radiology, Nuclear Medicine & Medical Imaging”, *Clinical Neuroradiology* is ranked 36th of 133 journals compared to 45th of 129 journals in the previous year.

The interest in articles published in *Clinical Neuroradiology* has also been steadily increasing. In 2019 articles in *Clinical Neuroradiology* were downloaded 69,893 times, compared to 63,347 times in 2018, and this trend persists. In the first 6 months of 2020, downloads have increased by 38.6% and submissions by 39.6%! Naturally, the increasing number of submissions means a higher workload for the Editorial Board. Nonetheless, the average peer review time has not increased. In 2019 the time from submission to first decision was 34 days.

These indicate our journal’s clear success. This success is primarily due to the excellent work of our reviewers and Editorial Board, who select and help shape the papers to be published with a keen sense for scientific quality and expertise in their fields. Not least thanks to their expert critique, the submitted works become all the more valuable to readers and scientists. We kindly thank all contributors for their valued support.

László Solymosi

Editor-in-Chief

